# A cell surface display fluorescent biosensor for measuring MMP14 activity in real-time

**DOI:** 10.1038/s41598-018-24080-0

**Published:** 2018-04-12

**Authors:** Alexander Braun, Matthew J. Farber, Zachary A. Klase, Peter B. Berget, Kenneth A. Myers

**Affiliations:** Department of Biological Sciences, University of the Sciences in Philadelphia, Philadelphia, PA 19104 USA

## Abstract

Despite numerous recent advances in imaging technologies, one continuing challenge for cell biologists and microscopists is the visualization and measurement of endogenous proteins as they function within living cells. Achieving this goal will provide a tool that investigators can use to associate cellular outcomes with the behavior and activity of many well-studied target proteins. Here, we describe the development of a plasmid-based fluorescent biosensor engineered to measure the location and activity of matrix metalloprotease-14 (MMP14). The biosensor design uses fluorogen-activating protein technology coupled with a MMP14-selective protease sequence to generate a binary, “switch-on” fluorescence reporter capable of measuring MMP14 location, activity, and temporal dynamics. The MMP14-fluorogen activating protein biosensor approach is applicable to both short and long-term imaging modalities and contains an adaptable module that can be used to study many membrane-bound proteases. This MMP14 biosensor promises to serve as a tool for the advancement of a broad range of investigations targeting MMP14 activity during cell migration in health and disease.

## Introduction

The advent of genetically encoded fluorescent proteins has revolutionized the field of cell biology, particularly in live-cell imaging. In recent years, there has been a boom in super-resolution imaging techniques that allow for nanoscale detection and localization of cellular proteins bound to fluorescent probes^1^. Despite these imaging advances, one continuing microscopy challenge is visualizing the activity state of proteins as a method to associate cellular outcomes with the behavior and activity of target proteins. Efforts to address this challenge have come a long way^2–9^, but caveats associated with the use of existing fluorescent probes, including spectral compatibility and spatio-temporal sensitivity, have limited the application of these biosensors to broader experimental investigations.

Fluorogen-activating proteins are single-chain variable fragments (scFv) of human antibodies that are able to bind non-fluorescent dye molecules and stabilize them in a fluorescent state^10^. These immunoglobulin-based fluoromodules cause a dramatic increase in fluorescence of the cognate dyes that they bind, the emission spectra for which is defined by the identity of the dye^11–13^. Excited-state dyes in solution undergo rotational and vibrational motions with non-radiative decay to the ground state, thus exhibiting very little fluorescence. However, upon binding to the fluorogen-activating proteins, conformational restriction is placed on the dye, thereby forcing relaxation to the ground state through radiative decay, with a large increase in fluorescence^11,12,14,15^. Fluorogen-activating proteins were first isolated from a human scFv library and thus consist of variable heavy (V_H_) and variable light (V_L_) chain domains connected by a flexible linker of [Gly_4_Ser]_*n*_ repeats^15–18^. Hybrid scFvs have been created by recombining the V_H_ and V_L_ domains of different fluorogen-activating proteins *in vitro*^13^. Through this work, it was discovered that certain fluorogen-activating proteins function as homodimers of variable domains, and that dye-binding can be blocked with specific domain combinations^19^.

Using fluorogen-activating protein technology, we have developed a biosensor selective for matrix metalloprotease 14 (MMP14; a.k.a. MT1-MMP). The biosensor was built by inserting a protease-selective target sequence between a dye-binding V_H_ domain and a blocking V_L_ domain to create a fusion protein that behaves as an MMP14 protease substrate. Cleavage of the protease target sequence releases the blocking domain, resulting in dye binding and dye fluorescence. MMP14 is a transmembrane, zinc metalloprotease that functions to remodel the extracellular matrix (ECM) during normal cell migration^20–25^. MMP14 activity is known to be globally upregulated during cancer metastasis^26–28^ and in other examples of aberrant cell motility^29–32^. Moreover, the function of MMP14 is thought to be highly regulated, such that both the localization and the activity of the enzyme can be adjusted by the cell in order to guide ECM remodeling and cell motility^33–38^. Given the wide biological significance of MMP14, there has been an increasing effort to develop tools capable of measuring MMP14 activity with the goal of understanding of how MMP14 function is controlled in both normal and diseased states^6,7,39,40^. Despite these efforts, to date there does not exist a methodology that enables dynamic measurements of MMP14 activity with high spatial and temporal resolution in living cells.

Here, we describe the design, validation, and live-cell implementation of a fluorescent biosensor capable of measuring MMP14 location, activity, and temporal dynamics, and that is applicable to both short and long-term imaging modalities. Our design not only advances the study of MMP14 activity in cellular migration in health and disease, but also provides an adaptable tool that can be used to study many membrane-bound proteases.

## Results

### Development of a fluorogen activating protein-based biosensor

**Figure 1:**
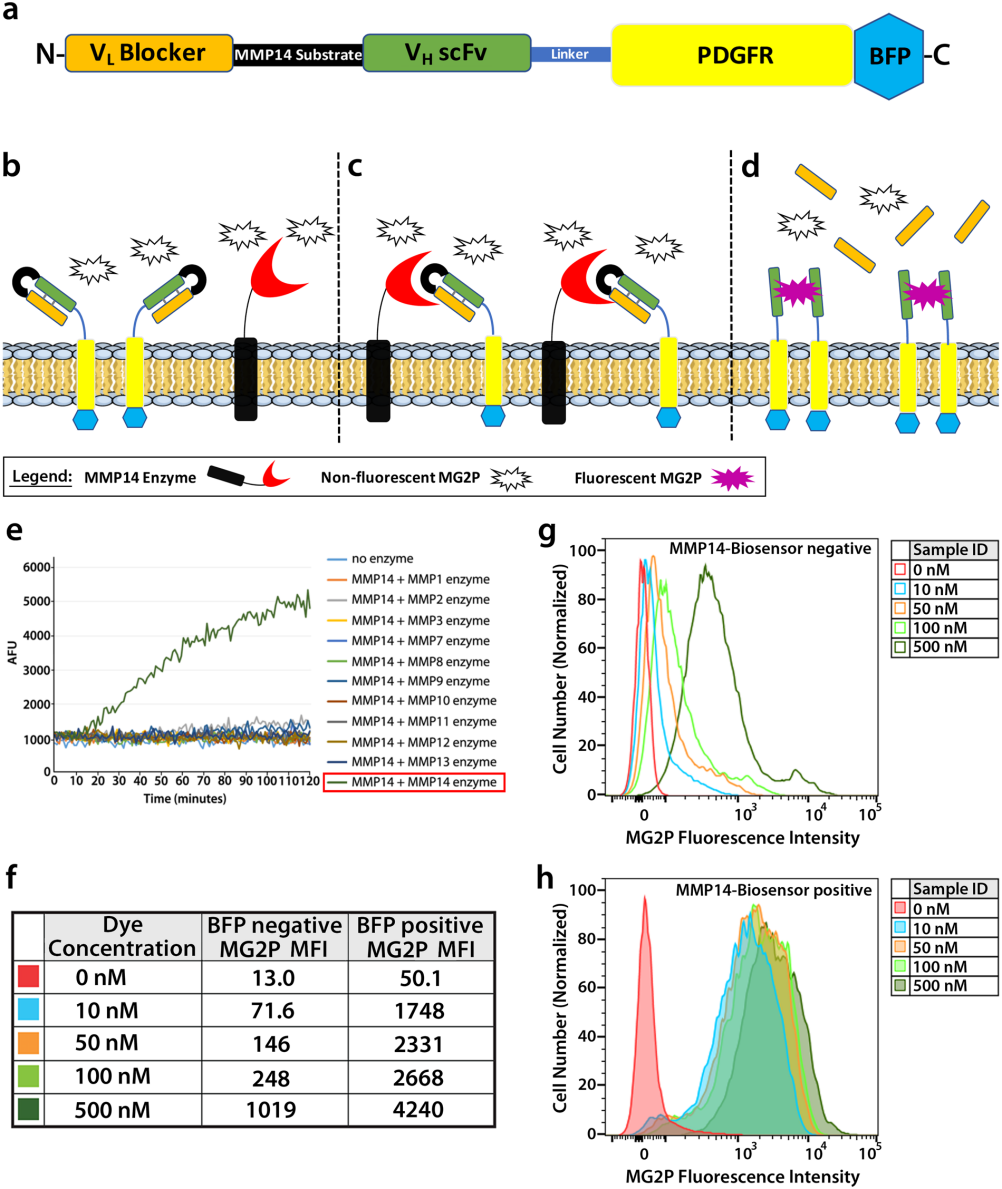
Development of a fluorogen activating protein-based biosensor. (**a**) Schematic diagram of the MMP14 biosensor subdomains and their relative positions (N-terminal to C-terminal; left to right) within the protein. (**b**–**d**) Schematic diagram showing the transmembrane MMP14 biosensor in the presence of MG2P dye when the biosensor is uncleaved (**b**), when the biosensor is in the process of being cleaved by the MMP14 enzyme (**c**), and when the cleaved MMP14 biosensor is bound to MG2P dye (**d**). (**e**) Tecan fluorimetry of soluble biosensor incubated with various catalytically active MMP enzymes showing specificity for MMP14. AFU = Arbitrary Fluorescence Units. (**f**–**h**) Cells were analyzed by flow cytometry to quantify total fluorescent signal from the biosensor. The level of MG2P fluorescence was determined for live BFP negative cells (**f**,**g**) or live BFP positive cells (**f**,**h**) and displayed as a histogram showing the number of cells versus MG2P fluorescence intensity (**g**,**h**). Color traces represent data from each of the different concentrations of dye used (0, 10, 50, 100 or 500 nM). MFI = Mean Fluorescence Intensity.

Labeling cell surfaces with fluorogen-activating proteins was first described in living cells in studies that characterized their interactions with different fluorogen derivatives^41^, and has since been engineered to detect various different targets primarily in live-cell systems^9,14,41–52^. Building from these findings, we set out to develop a fluorogen activating protein-based biosensor that would allow for visualization of MMP14 activity in real-time. The extracellular portion of the MMP14 biosensor consists of two scFv domains that flank a selective substrate sequence for MMP14 (Fig. 1a)^13,41,53^. The scFv domains fold in such a way that one acts as the binding partner of the MG2P dye, while the other acts as a blocker of that binding^13^ (Fig. 1b,c). The blocked hybrid scFv^13^ was modified by adding an MMP14-selective protease sequence between the V_H_ and V_L_ domains, and the modified DNA module was cloned into the pDisplay plasmid vector containing a mammalian CMV promoter (see Fig. 1 and Online Methods for details). The pDisplay plasmid vector contains a N-terminal IgK leader sequence that directs the fusion protein into the secretory pathway towards the cell surface, and a PDGFR transmembrane stop sequence to allow insertion of the biosensor into the plasma membrane. Once at the cell surface, interaction of active MMP14 enzyme with the MMP14 biosensor results in cleavage and release of the blocker, thereby allowing MG2P dye binding and fluorescence (Fig. 1d). MG2P is a cell-impermeable, malachite green derivative dye that fluoresces at the far-red wavelength when two V_H_ scFv domains dimerized around it. Using this approach creates a “switch-on’’ system, with no binding of dye to uncleaved biosensor^13^. Finally, we added the gene sequence for blue fluorescent protein (BFP) at the C-terminal, (cytoplasmic) portion of the cDNA to allow for detection of biosensor expression levels following transient transfection, and also to provide a method for monitoring biosensor location and dynamics.

An ongoing problem with studying specific MMPs is that they often share redundant substrates^54^. Therefore, it was imperative to find a sequence that was selective for MMP14. A six-amino acid sequence was used, ARGIKL, that was discovered as a substrate for MMP14 through a phage screen. This sequence was tested using a soluble version of the biosensor in a Tecan fluorimeter with eleven different MMPs, and was found to be specifically cleaved only by MMP14 (Fig. 1e).

To determine the saturation of MG2P dye binding to the MMP14 biosensor, flow cytometry was performed using a range of MG2P dye concentrations (Fig. 1f–h, and Figure S1). These experiments revealed that, in mock transfected endothelial cells (ECs; BFP negative) and in ECs expressing the MMP14 biosensor (BFP positive cells) the mean fluorescence intensity (MFI) was similar between both groups in the absence of MG2P dye (Fig. 1f). Addition of MG2P dye at concentrations of 10 nM, 50 nM, 100 nM, or 500 nM revealed that increasing the dye concentration resulted in increasing background fluorescence intensity (Fig. 1f,g). Analysis of MFI for each of the dye concentrations further revealed that dye binding to cells expressing the biosensor was similar at 10 nM, 50 nM, and 100 nM dye concentrations (Fig. 1f,h), while the background MFI increased 3.5-fold across this range (10 nM MFI = 71.6; 100 nM MFI = 248; Fig. 1f,h). Additionally, comparisons of MG2P fluorescence intensity in biosensor-positive ECs revealed a 24.4-fold MFI increase at dye concentrations of 10 nM, with the fold-change MFI consistently reduced at increasing concentrations of MG2P dye (50 nM = 16-fold; 100 nM = 10.8-fold; 500 nM = 4.2-fold). Thus, based on the outcomes of flow cytometry data, we chose to use 10 nM MG2P dye for all live-cell biosensor experiments.

### The MMP14 biosensor is trafficked through normal exocytic and endocytic pathways

**Figure 2:**
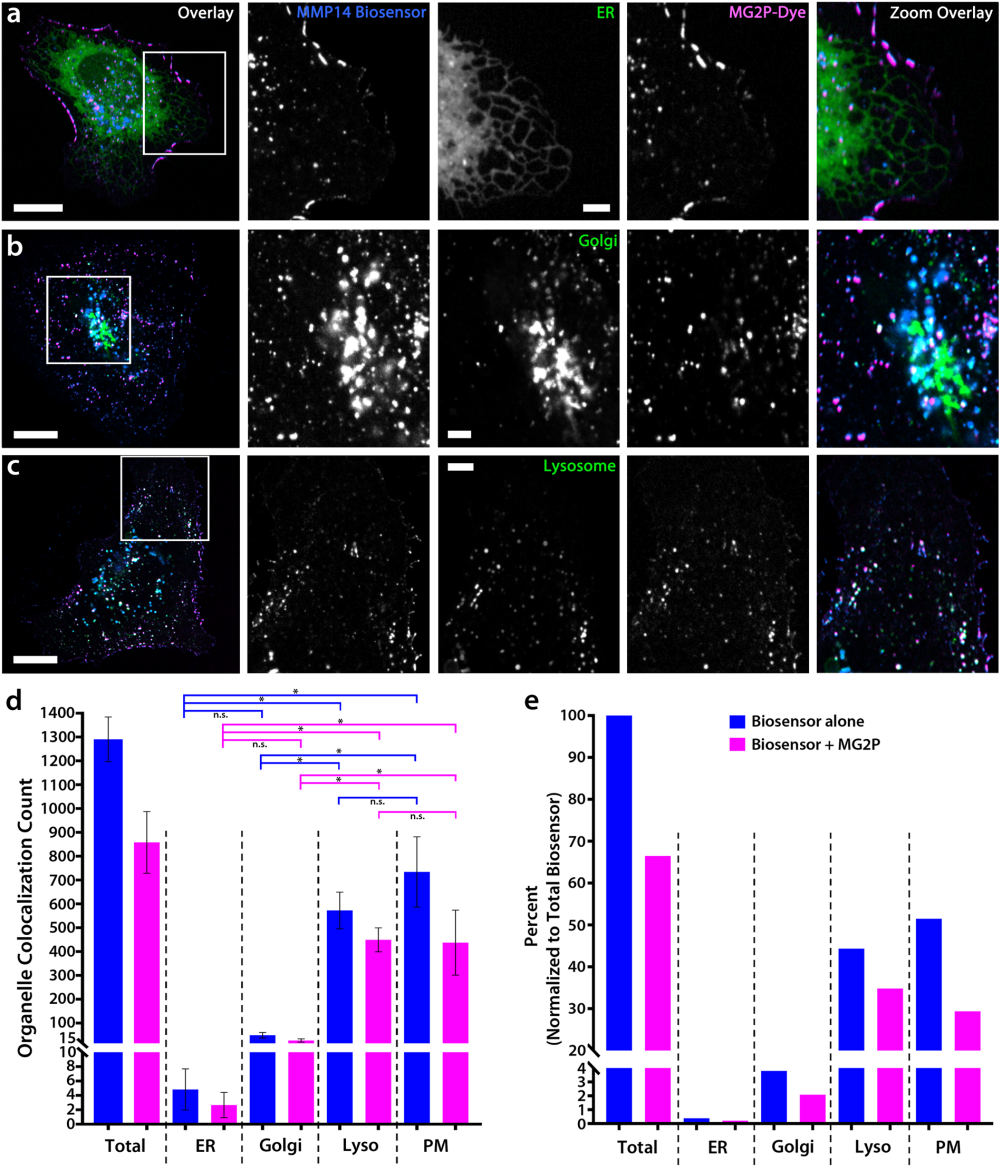
The MMP14 biosensor is trafficked through normal exocytic and endocytic pathways. (**a**–**c**) Whole-cell image overlays (far left) and zoomed regions of the cell (white boxes) showing colocalization imaging of HUVECs expressing the MMP14 biosensor, MG2P dye, and a fluorescent marker for either the endoplasmic reticulum (ER; **a**), the Golgi-apparatus (**b**), or lysosomes (**c**). The color of the text in each panel indicates the fluorescent color shown in the Overlay images. (**d**–**e**) Quantification of the total number of MMP14 biosensor and dye-bound biosensor and their distributions in ER (n = 6), Golgi (n = 5), lysosomes (n = 4), and the plasma membrane (PM; n = 15). (**e**) Distributions of MMP14 biosensor and dye-bound biosensor shown as a percentage of the total MMP14 biosensor localized within each of the cellular organelles. Scale bars = 20 µm (whole cell); 5 µm (zoomed). P < 0.01. Error bars =  + /− SD.

To evaluate MMP14 biosensor behavior in cells, we measured the localization and trafficking of BFP-biosensor and dye-bound biosensor in ECs expressing markers for cytoplasmic organelles including the endoplasmic reticulum (ER), Golgi apparatus, and lysosomal vesicles, using 2-dimensional colocalization imaging (Fig. 2). This outcome was further confirmed using 3-dimensional, z-stack colocalization imaging (Figure S2). These studies revealed minimal colocalization of the biosensor alone, or of the dye-bound biosensor, with the ER (biosensor = 0.37%, dye-bound biosensor = 0.21%; Fig. 2a,d,e). Colocalization measurements were slightly, but not significantly, increased in the Golgi apparatus compared to ER colocalization (biosensor = 3.80%, dye-bound biosensor = 2.09%; p = 0.9986; Fig. 2b,d,e). These results indicate that the biosensor trafficking through the trans-Golgi network to the plasma membrane (PM) occurs rapidly.

Analysis of colocalization with lysosomal structures revealed a significant increase in both the biosensor and dye-bound biosensor (p < 0.01; Fig. 2c and Video 1). Measurements revealed that 44.4% of total biosensor was colocalized with the lysosomes, while 34.8% of total biosensor was dye-bound within lysosomes (Fig. 2e). Thus, lysosome-associated biosensor was bound to MG2P dye 78.5% of the time. Because MG2P is impermeable to the cell, these results indicate that the biosensor was undergoing lysosomal endocytosis following biosensor cleavage and dye binding at the cell surface (Fig. 2c). Finally, measurements of biosensor colocalization revealed that the majority (51.5%) of total MMP14 biosensor was localized at the PM of ECs, while only 29.4% of total biosensor was dye-bound at the PM (Fig. 2e). These results highlight that 57.1% of the biosensor that localized at the PM was dye-bound, and suggest that a subpopulation of PM biosensor (42.9%) remained uncleaved, or cleaved and not dye-bound, at any particular moment in time.

To evaluate the potential for biosensor pre-cleavage prior to insertion at the cell surface, experiments using a fluorescent label specific for exocytic vesicles (Rab8a)^23^ were combined with the lysosomal marker and a cell-permable version of the MG2P dye (MG ester). These experiments revealed that Rab8a-labeled exocytic vesicles containing the MMP14 biosensor did not bind to the MG ester dye (Figure S3a,c). Additionally, expression of a fluorescently-labeled MMP14 enzyme revealed an absence of MG ester dye-binding in Rab8a-labeled exocytic vesicles containing both the MMP14 biosensor and the MMP14 enzyme (Figure S3b,c). The results of MG ester experiments show that the MMP14 biosensor is not pre-cleaved by endogenous MMP14, nor is the biosensor pre-cleaved in exocytic vesicles containing exogenously expressed MMP14 enzyme. Taken together, these results identify that the MMP14 biosensor is trafficked through normal exocytic and endocytic pathways and that the binding of MG2P dye is detected primarily at the PM, and in endocytic vesicles following biosensor activation and dye binding at the PM.

### MMP14 biosensor binding of MG2P dye on the EC surface is dynamic

**Figure 3:**
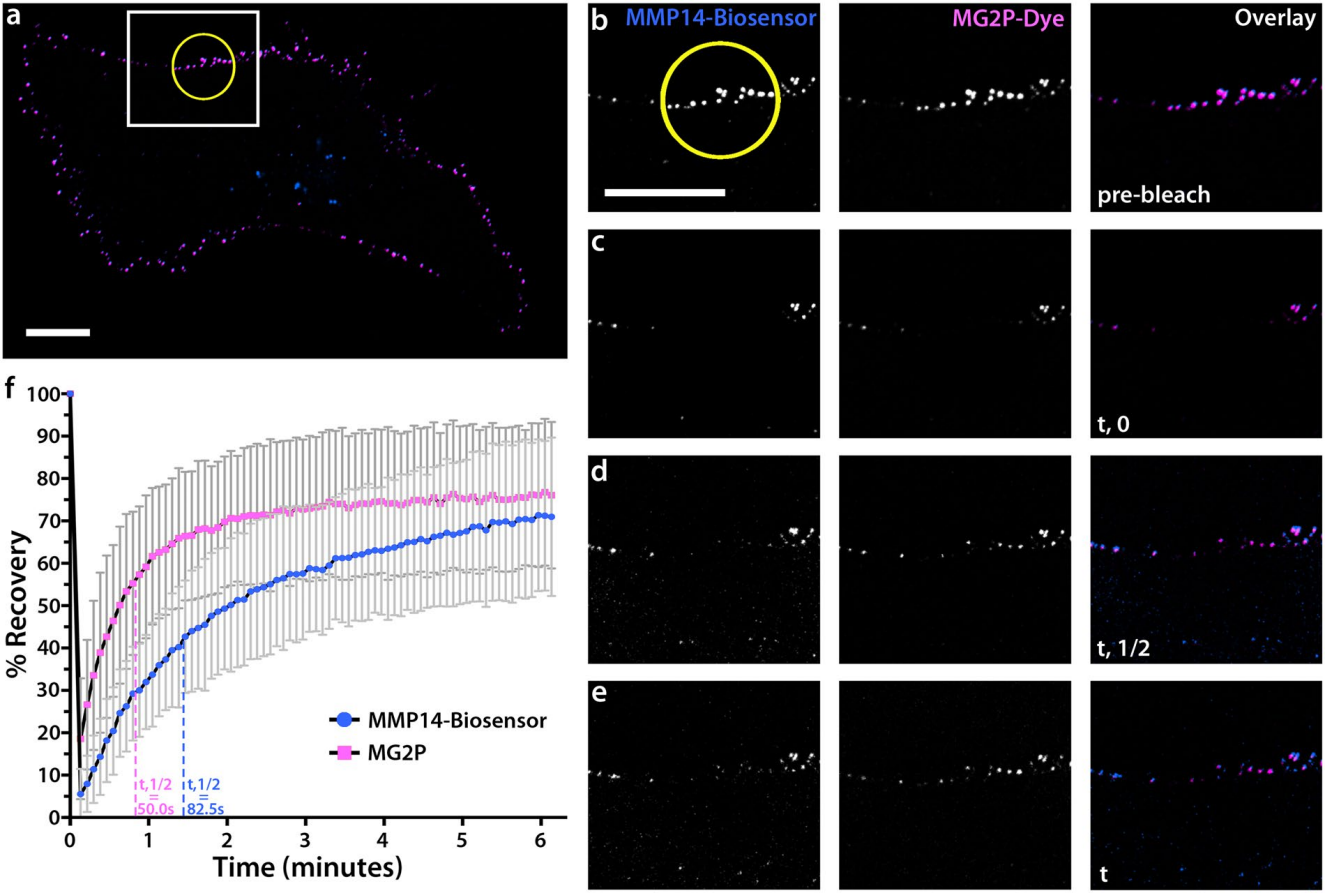
MMP14 biosensor binding of MG2P dye on the EC surface is dynamic. (**a**) Whole-cell image of an EC expressing MMP14 biosensor in the presence of MG2P dye. White box represents the zoom regions shown in (**b**–**e**). Yellow circle is the region of photobleaching. (**b**–**e**) Zoomed regions from (**a**) showing example grayscale images of MMP14 biosensor, MG2P dye, and Overlay at defined times during the FRAP experiment: (**b**), pre-bleach; (**c**), immediately following the photo-bleach (t, 0); (**d**), half time of final measured fluorescence recovery (t, ½); (**e**), total time of final measured fluorescence recovery (t). (**f**) Fluorescence recovery graph for the MG2P dye (pink) and the MMP14-biosensor (blue). Vertical dashed lines represent the position of t,1/2 for MG2P dye (pink) and the MMP14-biosensor (blue). Scale bars = 20 µm. Error bars =  + /− SD.

A previous investigation using fluorescence spectral measurements of MG2P dye dissociation from the H6 fluorogen-activating protein revealed dissociation constants in the low nanomolar range both for yeast surface-displayed scFvs and for soluble scFvs (7.5 nM surface Kd; 38 nM soluble Kd)^41^. These results highlight that fluorogen-activating protein dye-binding interactions are dynamic. We set out to determine the kinetics of biosensor and MG2P dye dissociation at the plasma membrane of living ECs using Förster (fluorescence) recovery after photobleaching (FRAP) microscopy experiments. To do this, we simultaneously photo-bleached a small region (10 µm in diameter) of the BFP-MMP14 biosensor and the MG2P dye-bound biosensor in ECs expressing the biosensor in the presence of MG2P dye, and acquired images at five-second intervals to determine fluorescence recovery rates. Image analysis revealed that both the biosensor and the biosensor-bound MG2P dye had undergone maximum fluorescence recovery within six minutes of photobleaching (Fig. 3 and Video 2), suggesting that both the biosensor and biosensor-bound MG2P dye turnover at rates that exceed those typical of endocytic or exocytic trafficking^55^.

Additionally, FRAP analysis revealed that the MG2P dye recovery occurred 1.65-fold faster than did the biosensor recovery (MG2P T_1/2_ = 50.0 seconds; BFP-biosensor T_1/2_ = 82.5 seconds; Fig. 3d,f). Measurements of the mobile and immobile fractions revealed that 55% of the biosensor was mobile compared with 60% of the dye, supporting reduced mobility of the BFP-biosensor compared to dye. These results show that the MG2P dye undergoes exchange with bleached biosensor, and that the majority of BFP-biosensor fluorescence recovery occurs via translocation of biosensor from non-bleached regions of the plasma membrane.

### Cleavage of the MMP14 biosensor requires functional MMP14 enzyme

**Figure 4:**
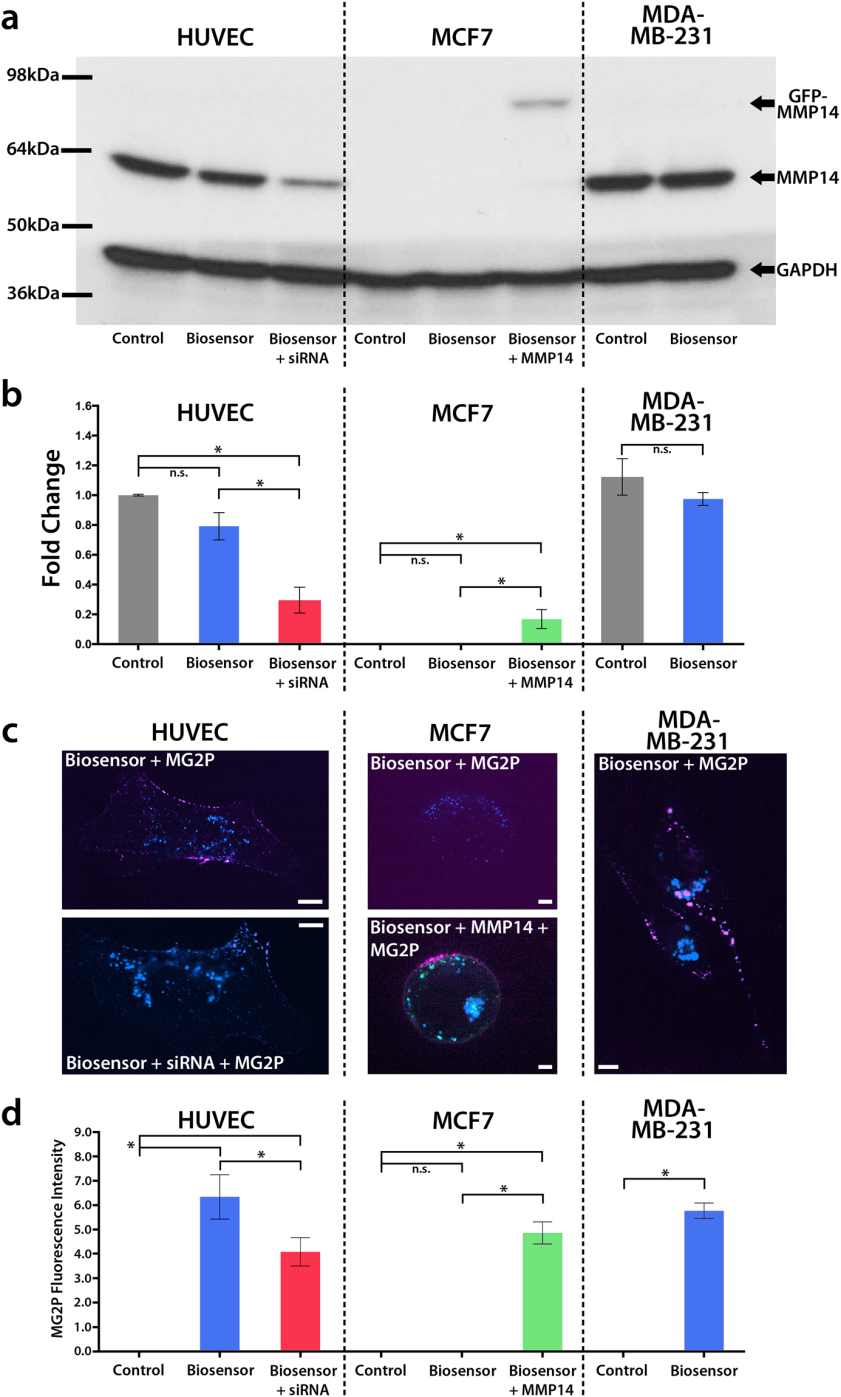
Cleavage of the biosensor requires functional MMP14. (**a**) Western blotting for GFP-MMP14, endogenous MMP14, and GAPDH in three different cell lines (HUVEC, MCF7, and MDA-MB-231). The cells were transfected with MMP14 biosensor prior to lysis. (**b**) Average densitometry measurements of western blots shown in (**a**) (n = 3). (**c**) Example confocal microscopy of HUVECs (n = 6), HUVECs + MMP14 siRNA (n = 3), MCF7 cells (n = 6), MCF7 + GFP-MMP14 (n = 3), and MDA-MB-231 cells (n = 3) showing MMP14 biosensor and MG2P dye binding under conditions shown in (**a**,**b**,**d**), Quantification of MG2P fluorescence intensity from the cells and conditions described in (**c**). Scale bars = 20 µm. P < 0.01. Error bars =  + /− SD.

*In vitro* Tecan fluorimeter investigations revealed that the MMP14 biosensor is cleaved by the MMP14 enzyme, and also showed that the MMP14 biosensor is not cleaved by any other MMPs that were tested (see Fig. 1e). We next set out to determine the specificity of the MMP14 biosensor for the MMP14 enzyme under conditions where the biosensor was expressed in living cells. To do this, we expressed the biosensor in three different cell lines: Human Umbilical Vein Endothelial Cells (HUVECs), MCF7 cells, a human breast adenocarcinoma cell line that does not express endogenous MMP14^56^, or MDA-MB-231 cells, a triple-negative human breast adenocarcinoma cell line with heightened MMP14 expression^57–61^. Because our primary experimental cell culture system uses HUVECs, all biochemical data were normalized to the HUVEC control condition. Western blot analysis revealed that expression of the biosensor caused a small, but statistically insignificant, reduction of MMP14 in HUVECs and MDA-MB-231 cells, and also confirmed that MCF7 cells do not express endogenous MMP14 (Fig. 4a,b). In HUVECs expressing the biosensor, MMP14 siRNA resulted in a significant reduction in MMP14 (Fig. 4a,b) and also resulted in significantly reduced biosensor-dye binding on the PM of HUVECs (Fig. 4c,d). MCF7 cells expressing the biosensor alone revealed biosensor-dye binding that was indistinguishable from background, while expression of exogenous GFP-MMP14 in MCF7 cells resulted in enhanced binding of the MG2P dye to the biosensor, further supporting the specificity of the biosensor for the MMP14 enzyme (Fig. 4c,d). Investigations of MDA-MB-231 cells revealed that MMP14 was slightly increased compared to HUVECs, and was not significantly affected by MMP14 biosensor expression (Fig. 4b). Addition of MG2P dye to the biosensor-expressing MDA-MB-231 cells revealed increased biosensor-dye binding compared to control, but was similar to biosensor-dye binding in HUVECs (Fig. 4c,d and Figure S4). Together, these data highlight that both the MMP14 biosensor and functional MMP14 enzyme are required to elicit MG2P dye fluorescence at the PM.

### The MMP14 biosensor is functional in tumor-cell and non-tumor cell environments

A large body of experimental evidence highlights that the tumor cell microenvironment is significantly more acidic than is the extracellular environment surrounding non-tumor cells. Published reports consistently document that the pH in tumor cell environments ranges from 6.1 to 7.4 (compared to the range of non-tumor cell pH from 6.8–7.4)^62–65^. It is important that the MMP14 biosensor be functional in cellular environments that encompass this range of pH. Thus, we set out to determine the pH dependence of MG2P dye-binding to the MMP14 biosensor. This analysis revealed that the MG2P dye binds to the MMP14 biosensor similarly across a range of pH, from 6.0 to 7.5, with a moderate reduction in dye-binding at pH = 5.5 and pH > 7.5, and significant loss of dye-binding at pH = 5.0 and lower (Figure S5). These data highlight that the MMP14 biosensor is functional both in non-tumor cell environments as well as in acidic environments common to tumor cells.

### MMP14 is locally activated in polarized ECs

Numerous investigations have shown that the activity of MMP14 is critical in driving directed migration of normal cell types, and also have identified that MMP14 is upregulated and aberrantly controlled in tumorigenic cell types^27,28,66,67^. In both normal and diseased states, MMP14 is known to promote directional cell migration through enzymatic digestion of the ECM^33,35–37,68–70^. This MMP-dependent activity facilitates the formation of new focal contacts between the cell and the ECM that enhance local remodeling of the matrix, and ultimately drives polarization of the cell including the formation of a leading and trailing edge^71–77^. We next set out to use the MMP14 biosensor in polarized, migrating ECs to determine if MMP14 activity is also polarized, as well as to measure the spatial and temporal kinetics of MMP14 activity during EC migration.

**Figure 5:**
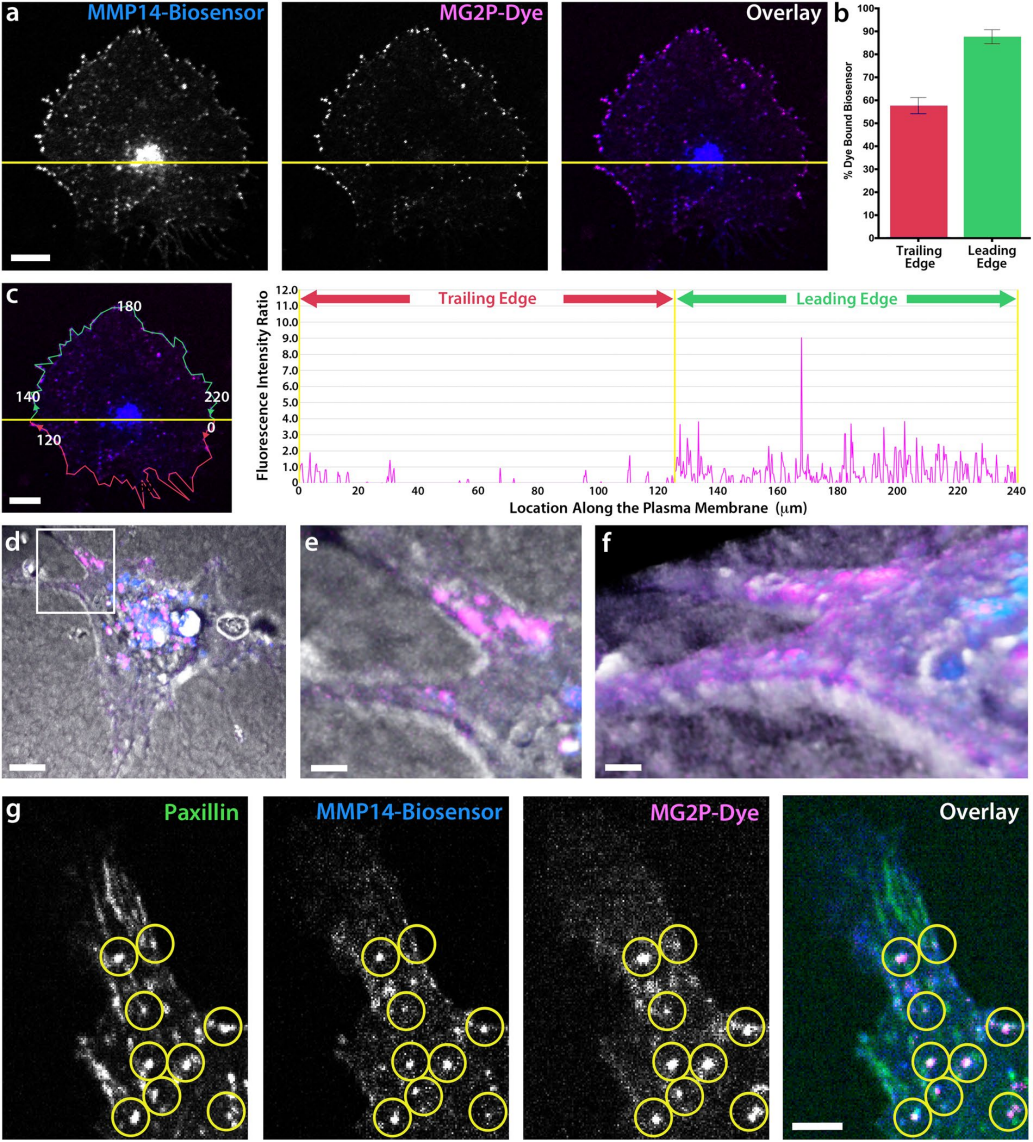
MMP14 is locally activated in polarized ECs. (**a**) Grayscale images of BFP-MMP14 biosensor, MG2P dye, and Overlay in a polarized HUVEC. The cell is divided by a yellow line into the leading edge (top) and trailing edge (bottom). (**b**) Quantification of the percent of dye-bound biosensor within the trailing edge and leading edge of ECs (n = 3). (**c**) Image of a cell (left panel) and intensity profile graph (right panel) showing the fluorescence intensity ratio of MMP14 biosensor and MG2P dye within the trailing edge and leading edge of the cell. Red line (left panel) is a cell perimeter mask of the trailing edge and green line (left panel) is a cell perimeter mask of the leading edge that is depicted in the intensity profile graph. Numerical values shown in (c, left panel), are provided for reference to the x-axis values within the intensity profile graph (right panel), which mark the location under the line (in microns) relative to the cell perimeter. (**d**–**f**) 3D z-stack whole cell image (**d**) showing a HUVEC expressing biosensor in a 3D type-I collagen ECM. Blue label is MMP14-Biosensor; Pink label is MG2P dye. (**e**) Zoomed image (white box in **d**) showing dye-bound biosensor within a branched protrusion at the interface of the cell and ECM, and a rotation of the same 3D zoomed image (**f**). (**g**) Grayscale images of a HUVEC branched protrusion showing a focal adhesion marker (GFP-Paxillin, left panel), BFP-MMP14 biosensor (left middle panel), MG2P dye (right middle panel), and Overlay (right panel). Yellow circles highlight focal adhesion structures that are colocalized with both the MMP14 biosensor and the MG2P-dye. (a,c,d) Scale bars = 20 µm. (e,f,g) Scale bars = 5 µm.

To do this we took two separate approaches. First, we conducted a migration assay of ECs cultured on a 2D type-I collagen ECM (see Online Methods for details) and imaged polarized ECs expressing biosensor that were actively migrating (Fig. 5a). MMP14 biosensor data revealed that the mean MG2P dye binding was significantly increased along the leading edge of the cell (Fig. 5b). Fluorescence intensity measurements of the ratio of dye-bound biosensor revealed a 3-fold average increase of MG2P dye binding to the biosensor within the leading edge, as compared with the trailing edge of the cell (Fig. 5c and Video 3). These data show that ECs have polarized MMP14 activity in the direction of cell migration (Fig. 5a–c).

In a second approach, we used the biosensor in ECs cultured in 3D type-I collagen sandwich gels^78^. Unlike their more spread morphology when cultured on 2D ECMs, in 3D ECMs, ECs display an elongated, spindle-like morphology that is driven by cytoskeletal reorganization resulting in branched protrusions that guide directional migration^79–91^. Confocal z-stack imaging of the biosensor in 3D collagen gels highlighted that the locations of the active, MG2P-bound biosensor were on the EC surface at positions in close in contact with the surrounding collagen ECM (Fig. 5d–f and Video 4). Punctate dye-bound biosensor was observed in 3D ECMs near the center of the cell, and this was qualitatively similar to lysosomal vesicles observed in our 2D analysis (see Fig. 2c). Using the 3D cell culture system, confocal imaging of ECs expressing the biosensor and GFP-paxillin, a fluorescent marker of focal adhesions, and treated with the MG2P dye, revealed that active MMP14 was localized to punctate structures directly adjacent to paxillin-labeled focal adhesions (Fig. 5g). Taken together, live-cell MMP14 biosensor imaging data directly identify that the activity of the MMP14 enzyme is spatially enhanced toward the leading edge of polarized ECs, and is spatially constricted near focal adhesion sites of cell-ECM engagement. To our knowledge, this is the first reported measurement of spatially and temporally distinct MMP14 activity in living ECs.

## Discussion

We have generated a MMP14-selective biosensor useful for detecting the localization and activity of endogenous MMP14 in migrating ECs in real time. This is the first MMP14 biosensor designed using a fluorogen activating protein-dye binding approach as a technology to specifically identify the locations on the cell membrane where MMP14 is active. Our data show that the biosensor is specifically cleaved by MMP14 and is not cleaved by other matrix metalloproteases, and that the biosensor is successfully delivered to the cell surface in its uncleaved form. Once at the cell surface, dye binding to the biosensor is dependent upon MMP14 cleavage of the biosensor, and dye binding to the biosensor is transient and dynamic. Finally, we used the biosensor to identify the location and dynamics of MMP14 activity in migrating ECs in 2D and 3D type-I collagen ECMs.

It is important to highlight the differences between this fluorogen-activating protein biosensor and previously published biosensors for matrix metalloproteases. The first description of a MMP14 biosensor was a FRET sensing element designed to measure MMP14 activity on the extracellular surface of polarized epithelial cells^75^. This same group has gone on to utilize the FRET biosensor to investigate functional aspects of MMP14 activity in several different biological contexts^5–7,39,40,75^. One limitation of FRET-based biosensors is the need to co-express donor and acceptor fluorescent probes that typically display similar emission spectra, resulting in reduced fluorescence emission intensities relative to background fluorescence. Because of this, the spectral profiles of selected fluorophores can affect the detection and interpretation of imaging data. Additionally, FRET biosensors must often be highly overexpressed to obtain measurable changes in donor/acceptor FRET intensities^8^, and this can have effects on normal cell physiology. The fluorogen-activating protein biosensor methodology eliminates the need to co-express donor and acceptor molecules, and thereby overcomes the limitations of signal detection. Moreover, because the MG2P dye is non-fluorescent prior to being bound to the cleaved biosensor, the background fluorescence of the system is nearly zero (with dye concentrations less than 100 nM; see Fig. 1f,g).

One characteristic of the FRET biosensor is the ability to see the biosensor in both the uncleaved as well as the cleaved state, due to the presence of donor and acceptor fluorophores on the probe. To achieve this with our biosensor, we introduced the C-terminal, intracellular BFP label that allows for visualization and tracking of the biosensor in both the inactivated as well as the activated (cleaved) form. Use of the BFP label for the biosensor is ideal for experimental applications using the MG2P dye, because it allows for clean separation of both fluorophores without the risk of spectral overlap. Additionally, unlike the FRET biosensor, the fluorogen-activating protein biosensor provides the potential for spectral separation of two additional fluorescent probes with wavelength emission profiles in the 490–660 nm range, commonly utilized in live cell imaging experiments to monitor the dynamics of labels such as GFP (peak emission = 509 nm) and mApple (peak emission = 592 nm).

Another, non-FRET-based, fluorogenic probe biosensor was reported to be capable of detecting the activity of matrix metalloproteases using fluorescence microscopy^92^. Similar to both the MMP14 FRET biosensor and our biosensor, this fluorogenic probe becomes fluorescent following protease-dependent cleavage of a substrate sequence engineered within the biosensor. Unlike the FRET and our fluorogen-activating protein biosensor, this fluorogenic probe is not displayed on the cell surface, but rather is attached to the ECM via chemical crosslinking, followed by the culture of live cells onto the biosensor-containing substrate. Thus, one advantage of the fluorogenic probe is that it does not require transfection of cells in order for the probe to work, making this a viable option for cell types that are not particularly amenable to cDNA expression.

A limitation of the ECM fluorogenic probe is that it displays promiscuity for MMP2, MMP9, and MMP14, making it difficult to measure protease activity that is specific to MMP14. Additionally, because the fluorogenic probe is directly conjugated to the ECM, it remains fluorescent indefinitely once cleaved, and thus acts as a static reporter of MMP activity. This limitation is likely to be noticed in studies of randomly migrating cells that move over an area of biosensor that has already been cleaved, or when conducting subcellular detection and analysis of MMP activity. The fluorogen-activating protein biosensor design overcomes the fluorogenic probe’s limitations because each molecule of biosensor is dynamically exchanged at the plasma membrane of the cell (T_1/2_ = 82.5 secs; see Fig. 3), allowing for high spatial and temporal resolution of MMP14 specific activity. Additionally, the unique design of the biosensor includes flexibility to engineer additional cleavage sequences that are specific for any number of proteases, thereby enhancing the utility of the biosensor as an adaptable tool for measuring the dynamics and activity of a wide array of cell-surface protease targets in living cells.

## Materials and Methods

### Cell Culture

Primary Human Umbilical Vein Endothelial Cells (HUVECs) were purchased (Lonza Inc.; Basel, Switzerland) and cultured in Endothelial Cell Basal Medium (EBM) medium supplemented with EGM-MV Single Quots (Lonza Inc.; Basel, Switzerland) and penicillin/streptomycin at 37 °C in 5% CO_2_ as previously described^93^. Michigan Cancer Foundation-7 cells (MCF7) were purchased (ATCC; Manassas, VA) and cultured in Eagle’s Minimum Essential Medium (MEM) supplemented with 0.01 mg/mL insulin and 10% fetal bovine serum (FBS) and penicillin/streptomycin at 37 °C in 5% CO_2_. Triple negative breast cancer cells (MDA-MB-231) were purchased (ATCC; Manassas, VA) and cultured in DMEM media supplemented with 10% FBS and penicillin/streptomycin at 37 °C in 5% CO_2_. For live imaging, HUVECs were cultured at 100,000 cells/dish on 10 µg/mL fibronectin coated 35 mm glass-bottom dishes (Cellvis, cat#: D35-20-1.5-N) and medium was supplemented with 25 mM  HEPES, pH = 7.2. Transfection of cDNAs was performed using a Lonza Nucleofector Device with solution kit V (Lonza), setting A-034 for HUVECs, P-020 for MCF7 cells, and X-001 for MDA-MB-231 cells. Experiments were performed 3–4 hours post-transfection to allow time for cDNA expression.

### MMP14 Protease-activated Cell-surface Fluorescent Biosensor

The MMP14 protease-activated cell-surface display biosensor was a modification of the soluble MMP14 biosensor created by P.B.Berget at Carnegie Mellon University and Enzium, Inc. For biosensor display on the cell surface, the biosensor DNA construct was cloned into the pDisplay mammalian expression vector (Thermo Fisher Scientific, cat#: V66020). The resulting protein fusion construct is driven by the CMV promoter and the murine Ig κ-chain leader sequence that directs the fusion protein into the secretory pathway and towards the cell surface. Following the biosensor sequence, the platelet-derived growth factor receptor (PDGFR) transmembrane domain anchors the fusion protein on the plasma membrane.

The original MMP14 biosensor construct was modified by swapping N-terminal H6 V_H_ fluorogen-activating protein domain and the C-terminal scFv1 V_L_ inhibitory domain (L1 V_L_) so that the H6 V_H_ domain was located proximal to the C-terminus of the fusion protein^13^. A MMP14 substrate sequence was engineered between the L1 V_L_ and the H6 V_H_ to contain 2 repeats of a (Gly_4_Ser) sequence followed by the MMP14 target sequence (Ala-Arg-Gly-Ile-Lys-Leu) followed by 3 repeats of (Gly_4_Ser). The modified construct was then PCR amplified with tailed ends for cloning into the pDisplay vector. To allow for biosensor localization, visualization, and measurement of biosensor expression, an mTag-BFP2 cassette was inserted into the pDisplay vector at a position C-terminal to the PDGFR domain, using primers containing BmgBI and XhoI restriction sites. This resulted in a modified pDisplay plasmid containing an intracellular mTag-BFP2, a single pass transmembrane domain, and an extracellular MMP14 protease selective biosensor. Upon proteolytic cleavage of the MMP14 selective sequence, the inhibitory L1 V_L_ domain is released and the H6 V_H_ domain remains tethered to the cell surface, where it dimerizes around MG2P dye, and induces fluorescence (see Fig. 1). The malachite green derivative, MG2P (se-Red-xc; SharpEdge labs), is a cell-impermeable dye that fluoresces when bound by a variety of different fluorogen activating proteins. In this application, the dye fluoresces when bound by two H6 V_H_ fluorogen activating proteins on the cell membrane. Fluorimetry of the soluble version of the modified MMP14 biosensor and MG2P dye (concentration = 500 nM) was performed using a Tecan fluorimeter testing various catalytically active human MMPs in solution (Enzo Life Sciences; Farmingdale, NY) to determine substrate specificity for MMP14.

### 3D Cell Culture

Rat tail type-I collagen at 3 mg/mL was used for all 3D cell culture experiments (Corning, cat#: 354249). The collagen mixture was made on ice containing: collagen I, MEM, H_2_O, and 7.5% NaHCO_3_ to create a 3D collagen “sandwich gel” as previously described^78^. 100,000 HUVECs were transfected with cDNA construct(s) and plated on to 35 mm cell culture dishes, and placed in the 37 °C incubator for 2 h to eliminate cells that did not survive transfection. A thin layer of the collagen mixture (25 µl) was spread onto a 35 mm-glass bottom dish and placed in the incubator for 30 min to polymerize. The transfected HUVECs were trypsinized after 2 h, plated onto the 25 µl collagen layer, and allowed to adhere to the collagen in the incubator for 1 h. 150 µl of the collagen mixture was then added on top of the cells and placed in the incubator for  2 h. Finally, 2 mL of EBM media was added to the dish prior to imaging.

### Microscopy

#### Hardware

Microscopic imaging was performed on a spinning disk (Yokagawa CSU-X1; Andor Technology) confocal microscope using a 60 × 1.4 NA oil immersion or a 60 × 1.2 NA water immersion objective lens on a TiE microscope equipped with Perfect Focus System (Nikon) equipped with an electronic shutter (Sutter Instrument) for transmitted illumination, a linear encoded X and Y, motorized stage (ASI Technologies), and a multi-bandpass dichromatic mirror (Semrock) and bandpass filters (Chroma Technology Corp.) in an electronic filterwheel for selection of BFP, GFP, or RFP emission. 405-, 488-, 561-, and 640-nm laser illumination was provided by a high-powered (20 mW 405-nm; 50 mW 488-; 561 and 640-nm) monolithic laser combiner module (MLC 400B; Agilent Technologies) that were shuttered with electronic shutters and directed to a fiber-coupled output port with an Acousto optic tunable filter and to the confocal scan-head via a singlemode polarization-maintaining fiber coupled delivery system (Agilent Technologies). Images were acquired using a Clara cooled CCD camera (Andor) operated in the 14-bit mode. Microscope system automation was controlled with NIS elements software (Nikon Instruments).

#### Live-cell MG2P and MG Ester Experiments

For all biosensor dye-binding experiments, 10 nM MG2P dye was added to the cells immediately prior to imaging. A cell line that does not express MMP14 endogenously, (MCF7 cells) and a malignant cancer cell line (MDA-MB-231) were transfected with the MMP14 biosensor  3 h prior to addition of MG2P dye and imaging for 2 min at 2 sec image intervals (Fig. 4). To determine parameters related to vesicular trafficking of the MMP14 biosensor and MG2P dye to and from the cell surface, HUVECs were transfected with mApple-LAMP1 (a lysosomal marker), mApple-B4GALT1 (a Golgi marker), and mApple-CALR (an endoplasmic reticulum marker) 4 h prior to addition of MG2P dye and imaging. For vesicular trafficking experiments, time-lapse images were acquired at 2 sec intervals for 4 min. To measure biosensor pre-cleavage, HUVECs were transfected with the mApple-LAMP1 lysosomal marker and GFP-Rab8a (an exocytic vesicle marker), or, they were transfected with GFP-Rab8a and mCherry-MMP14, 4 h prior to addition of a cell-permeable dye (MG ester) and imaging.

#### FRAP

Förster (Fluorescence) recovery after photobleaching (FRAP) experiments were performed using a Nikon LU-N4 compact laser unit equipped with four lasers (405-, 488-, 561-, and 640 nm), each with an output power of 15 mW. The laser unit was combined with the Nikon TiE spinning disk microscope previously described. Experiments were performed by first identifying a region of interest (ROI) on the cell surface containing both active and non-active biosensor. The ROI was bleached using 90% laser power with a 350 µs dwell time using the 405 nm laser for the biosensor and the 640 nm laser for MG2P dye. Images were acquired post-FRAP at five second intervals for a total of 6 minutes. Fluorescence recovery was quantified for both the biosensor and the MG2P dye over the indicated time course. The halftime of recovery was calculated as the time from the bleach to the time point where the fluorescence intensity reaches the half (*t*_1/2_) of the final recovered intensity (*F*_∞_)^94^.

### Colocalization Image Analysis

#### Dye-binding Intensity

For colocalization analysis of polarized, migrating HUVECs, cells were selected and the direction of cell migration was determined using live-cell, time-lapse imaging. From the time-lapse images, individual frames were binarized for each fluorescent channel (MMP14-Biosensor and MG2P dye). All images were background subtracted using a Region Of Interest (ROI) within the field of view. The cell centroid was then calculated (using NIS Elements software) and a yellow line was drawn perpendicular to the direction of migration, and through the centroid, to define the leading and trailing edge of the cell. A second line (1 µm width) was drawn to mask the cell periphery, beginning at the yellow centroid line marking the start of the trailing edge, and ending at the same position by tracing the entire cell periphery. The ratio of the fluorescence intensity of MG2P to MMP14 biosensor was calculated under the line, and was plotted relative to the fluorescence location along the plasma membrane (µm).

For MG2P colocalization analysis comparisons between cell types, measurements of MG2P dye binding intensities for HUVECs, HUVECs + MMP14 siRNA, MCF7 cells, MCF7 + GFP-MMP14, and MDA-MB-231 cells were calculated by first subtracting a background ROI for each cell. A 1 µm wide line was drawn to mask the entire cell periphery. Fluorescence intensities of the MG2P-dye were measured under the line and the relative mean intensities for each cell were quantified.

### 3-Dimensional Colocalization Analysis

Colocalization analysis of vesicular trafficking was performed following transfecting of HUVECs with mApple-LAMP1 (a lysosomal marker), mApple-B4GALT1 (a Golgi marker), and mApple-CALR (an endoplasmic reticulum marker) 4 h prior to addition of MG2P dye and imaging. 3D images were obtained at 0.3 micron steps ± 5 microns from the focal plane (total Z distance = 10 microns). Images are presented in slice view, consisting of a reticule to visualize colocalization in both xz and yz planes along the cell. Orange crosshairs in the whole cell image mark the subcellular structures shown in the xz and yz images.

### Flow Cytometry

HUVECs cells were cultured as described above. Cells were transfected with either no DNA (mock) or with MMP14 biosensor and plated onto 60 mm culture dishes. After 5 h, MG2P dye was added to the dishes at final concentrations of 10-, 50-, 100- or 500 nM in 2 mL of EBM media. At 6 h total time, the media was aspirated off and 500 µL of PBS was added to each dish. The MG2P dye concentrations were maintained in the PBS. Cells were then scraped off the dish, dissociated *via* manual trituration, and placed into falcon tubes on ice for flow cytometry readings. Flow cytometric determination of blue fluorescent protein expression and MG2P fluorescence was performed on a Cytek DxP12 flow cytometer utilizing the 407 nm laser with 450/50 bandpass detector for blue fluorescent protein and the 637 nm laser with 661/16 bandpass filter for MG2P. Live cell populations were gated on FSC by SSC. Data analysis was performed for n = 100,000 cells.

### pH Measurements

The H6 V_H_ fluorogen activating protein was expressed and purified as previously described (13). To test the effect on pH, purified H6 was added to equimolar MG2P in sodium acetate (pH 4.0–5.5), PBS (pH 6.0–7.0), or Tris (pH 7.5–9.0) in the presence of 0.2% Pluronic F-127 (Sigma Aldrich). After incubation for 10 minutes, endpoint RFUs were measured with excitation at 635 nm and emission at 660 nm via a Tecan infinite M1000.

### Biochemistry

Cytoplasmic extracts from HUVEC, MCF7, and MDA-MB-231 cells were isolated in NP40 lysis buffer (50 mM Tris, pH = 8.0, 150 mM NaCl, 0.5% Sodium Deoxycholate) supplemented with protease inhibitor cocktail (Thermo Scientific, cat #: 78430). Following lysis, cell nuclei were removed via centrifugation. Proteins from supernatants were quantified by the Bradford method. 10 µg of protein was mixed in Laemmli sample buffer (Bio-Rad, cat #: 161–747) and separated by SDS-PAGE. After electrophoresis, proteins were electrophoretically transferred to an immobilon-P membrane. For protein detection, membranes were blocked for 1 h at room temperature with 4% BSA in TBS-T buffer (20 mM Tris, pH = 7.6, 137 mM NaCl, and 0.1% Tween-20) and incubated overnight at 4 °C with rabbit anti-MMP14 (1:2500, Abcam, cat #: ab51074) and mouse anti-GAPDH (1:2500, Abcam, cat#: ab9484). After primary antibody incubation, blots were washed five times with TBS-T (5 min each) and incubated with appropriate HRP-conjugated secondary antibodies (1:40000; Jackson Immunoresearch) for 1 h at 37 °C. Blots were washed five times (5 min each) with TBS-T and protein bands were visualized using an ECL detection system (Pierce, SuperSignal West Pico Chemiluminescent Substrate). For protein densitometry measurements, western blot protein bands were normalized to respective GAPDH loading controls, followed by measurements of the normalized digital images of immunoblot bands using ImageJ64 software (NIH) as previously described^95^.

### Statistical analysis

All statistical analyses were performed on Graphpad Prism software using a one-way ANOVA with Tukey’s multiple comparison test with statistical significance set to P < 0.01.

### Data availability

The authors declare that data generated or analyzed during this study are included in this published article (and its supplementary information files).

## Electronic supplementary material

Supplementary Information

Video 1

Video 2

Video 3

Video4
