# Supplementary material for: A cell surface display fluorescent biosensor for measuring MMP14 activity in real-time
Source: Sci Rep. 2018 Apr 12;8:5916. doi: 10.1038/s41598-018-24080-0 (PMC5897415; doi:10.1038/s41598-018-24080-0)
Supplement: Supplementary file 1 — Supplementary Information [file 41598_2018_24080_MOESM1_ESM.pdf]

Supplementary Materials for Manuscript: SREP-17-49217A

Title: A cell surface display fluorescent biosensor for measuring MMP14 activity in real-time.

Authors: Alexander Braun[1], Matthew J. Farber[1], Zachary A. Klase[1], Peter B. Berget[1], and Kenneth A. Myers[1]

[1]Department of Biological Sciences, University of the Sciences in Philadelphia, Philadelphia PA 19104

Correspondence:

Kenneth A. Myers

Department of Biological Sciences

600 S 43rd Street

Philadelphia, Pennsylvania 19104

Phone: 215-596-7531

Fax: 215-596-8710

Email: k.myers@usciences.edu

Running Header: A fluorogen activating protein biosensor for MMP14 activity.

Figure S1

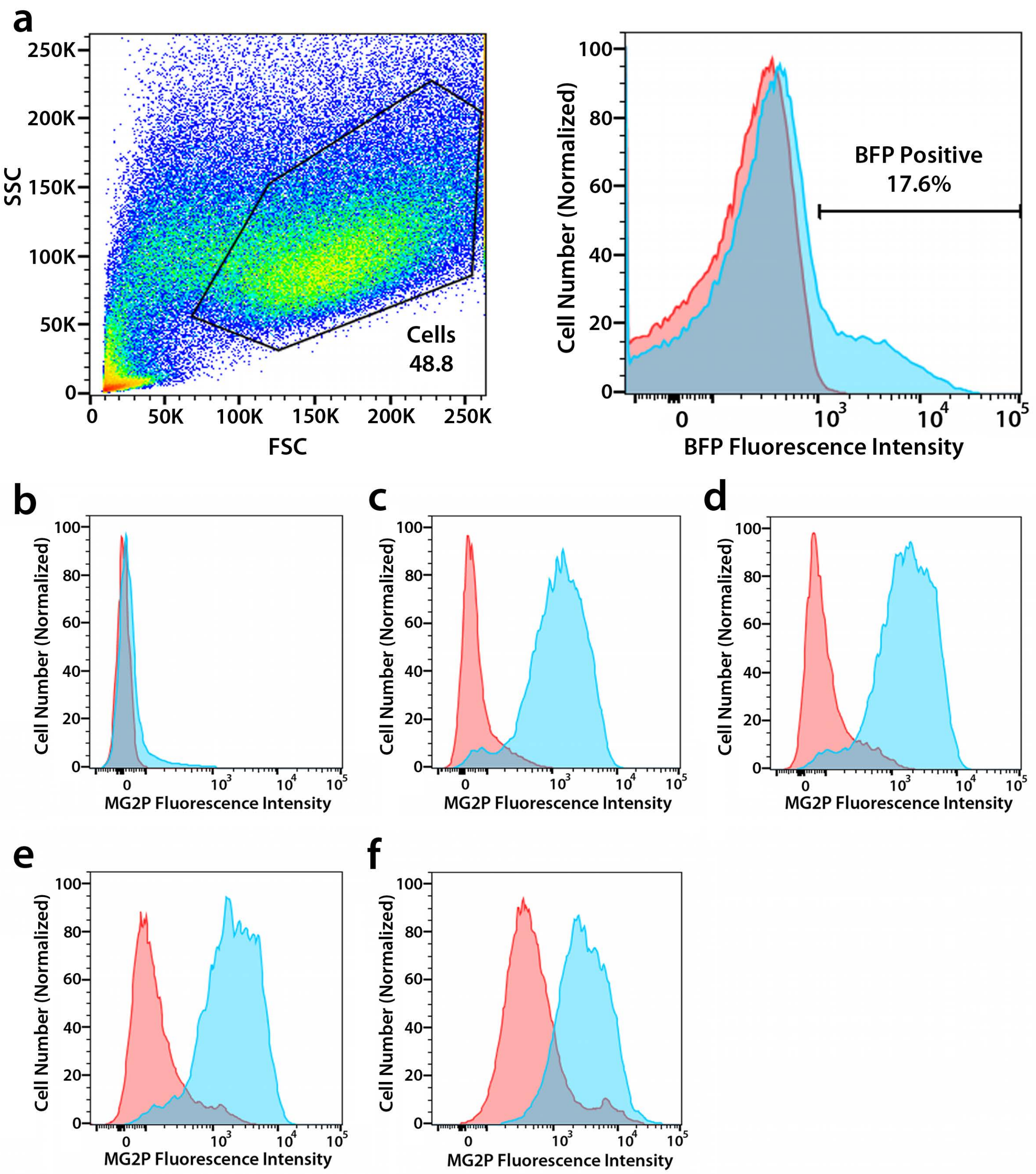

a
SSC
FSC
250K
200K
150K
100K
50K
0
Cells
48.8
Cell Number (Normalized)
BFP Fluorescence Intensity
BFP Positive
17.6%
b
c
d
e
f
MG2P Fluorescence Intensity

Figure S2

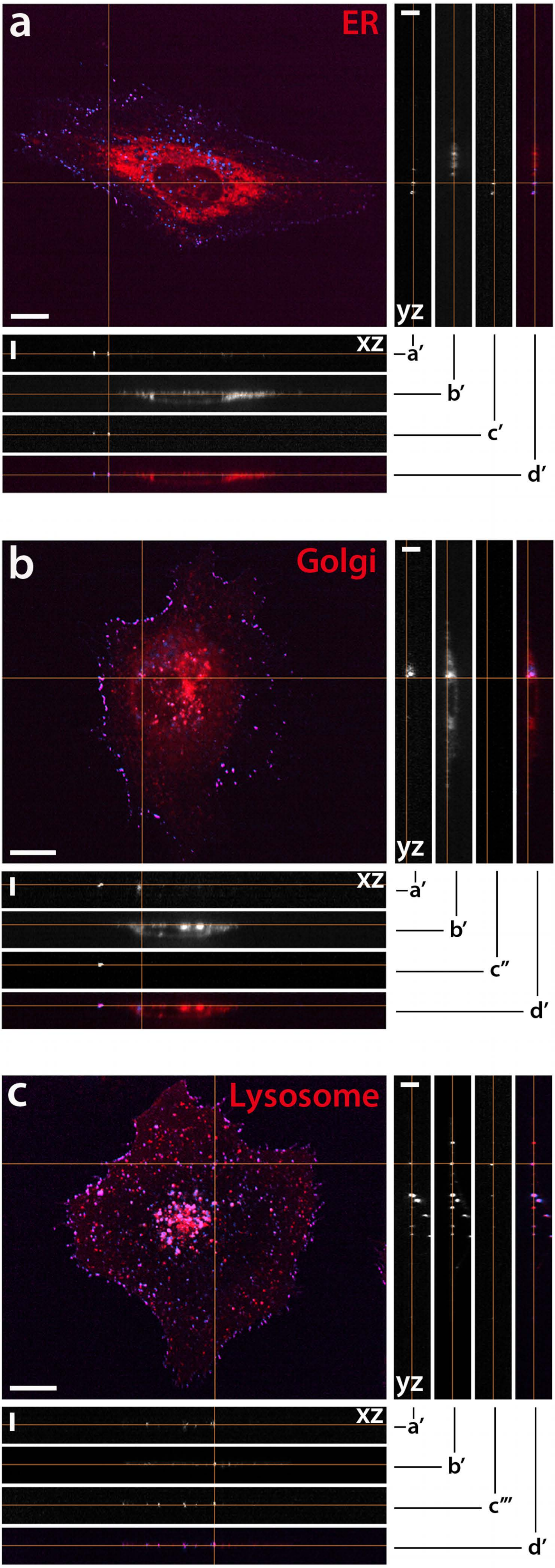

Figure S3

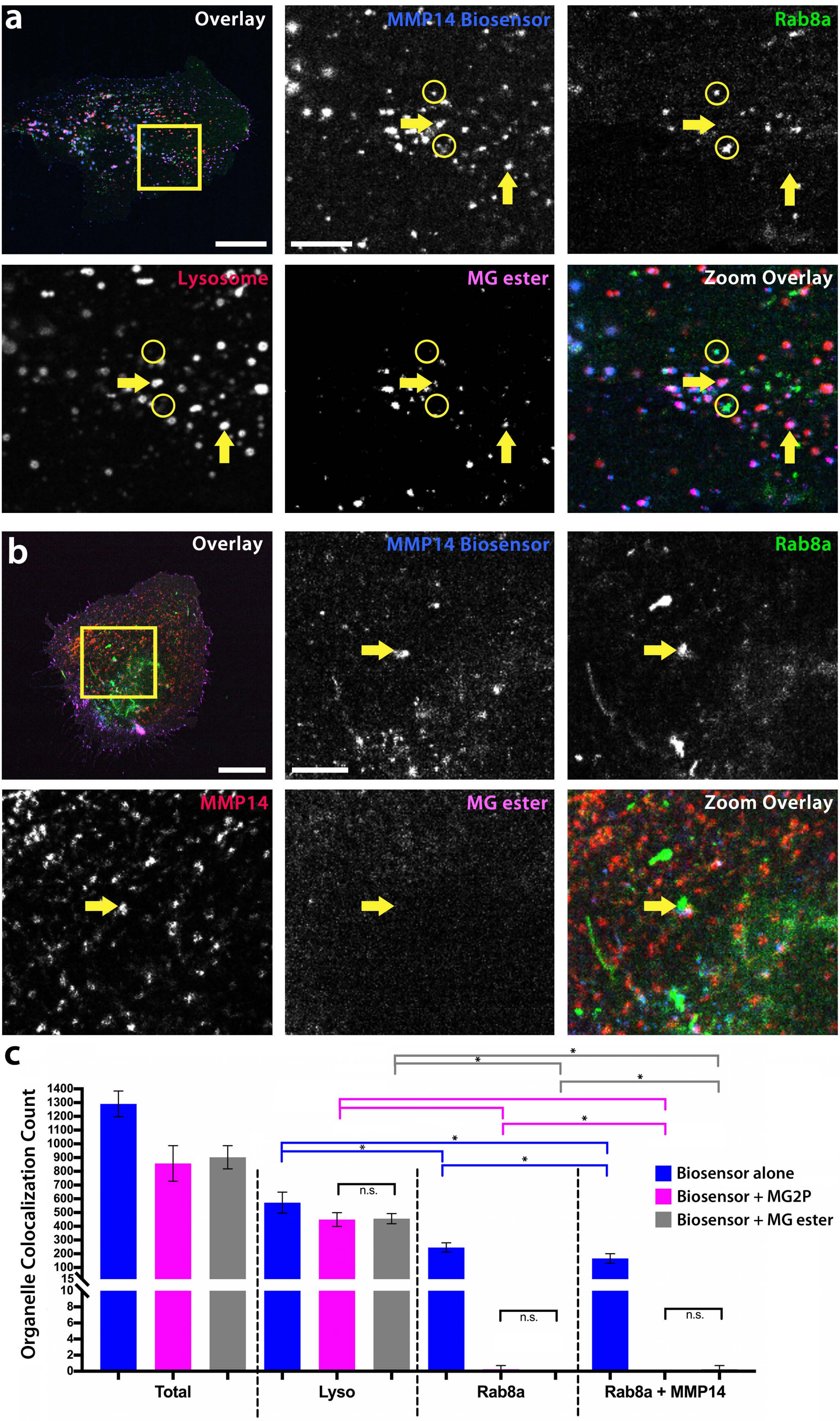
a
Overlay
MMP14 Biosensor
Rab8a
Lysosome
MG ester
Zoom Overlay
b
Overlay
MMP14 Biosensor
Rab8a
MMP14
MG ester
Zoom Overlay
c
Organelle Colocalization Count
1400
1300
1200
1100
1000
900
800
700
600
500
400
300
200
100
15
10
8
6
4
2
0
n.s.
*
Biosensor alone
Biosensor + MG2P
Biosensor + MG ester
Total
Lyso
Rab8a
Rab8a + MMP14

Figure S4

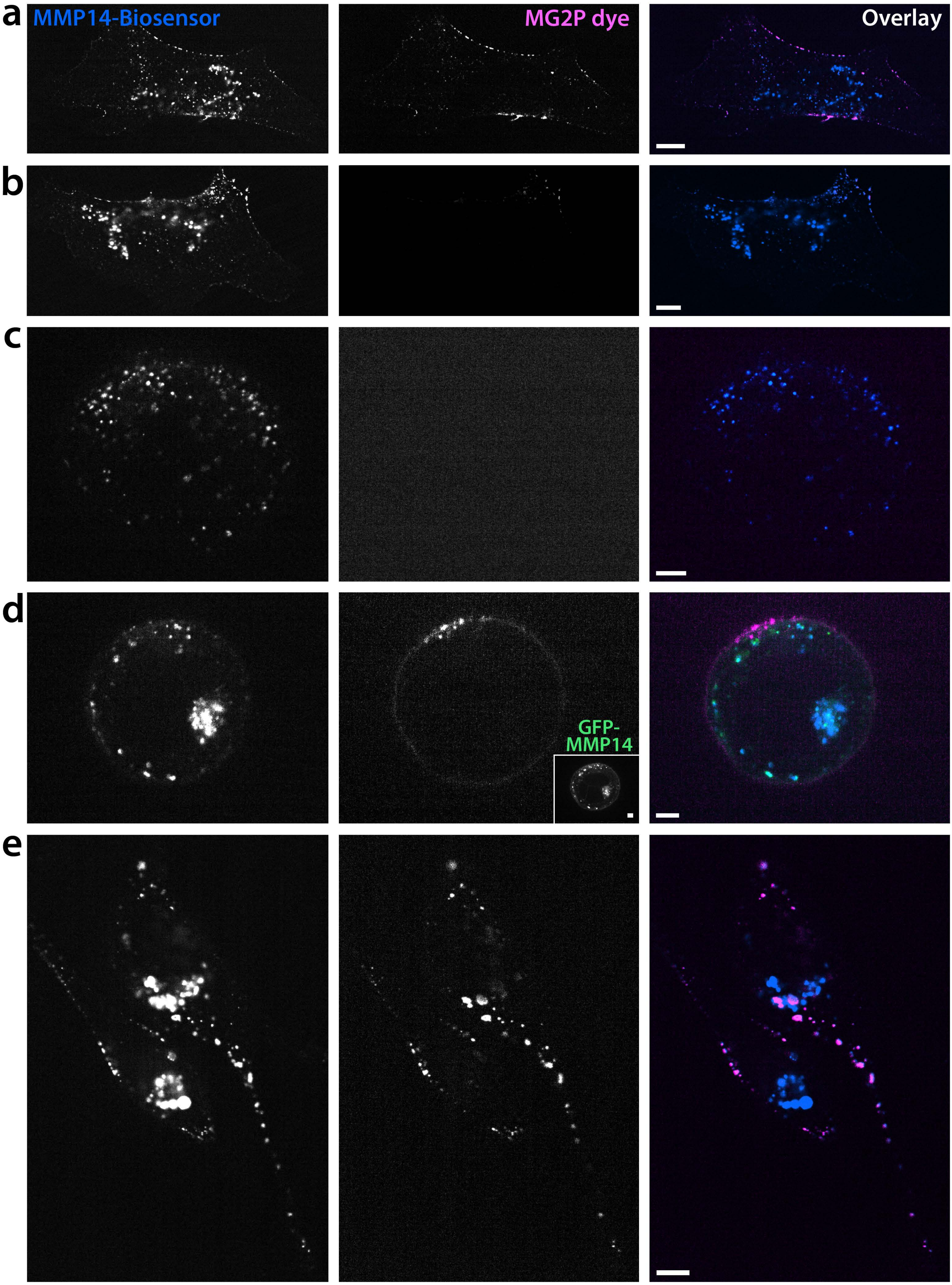

Figure S5

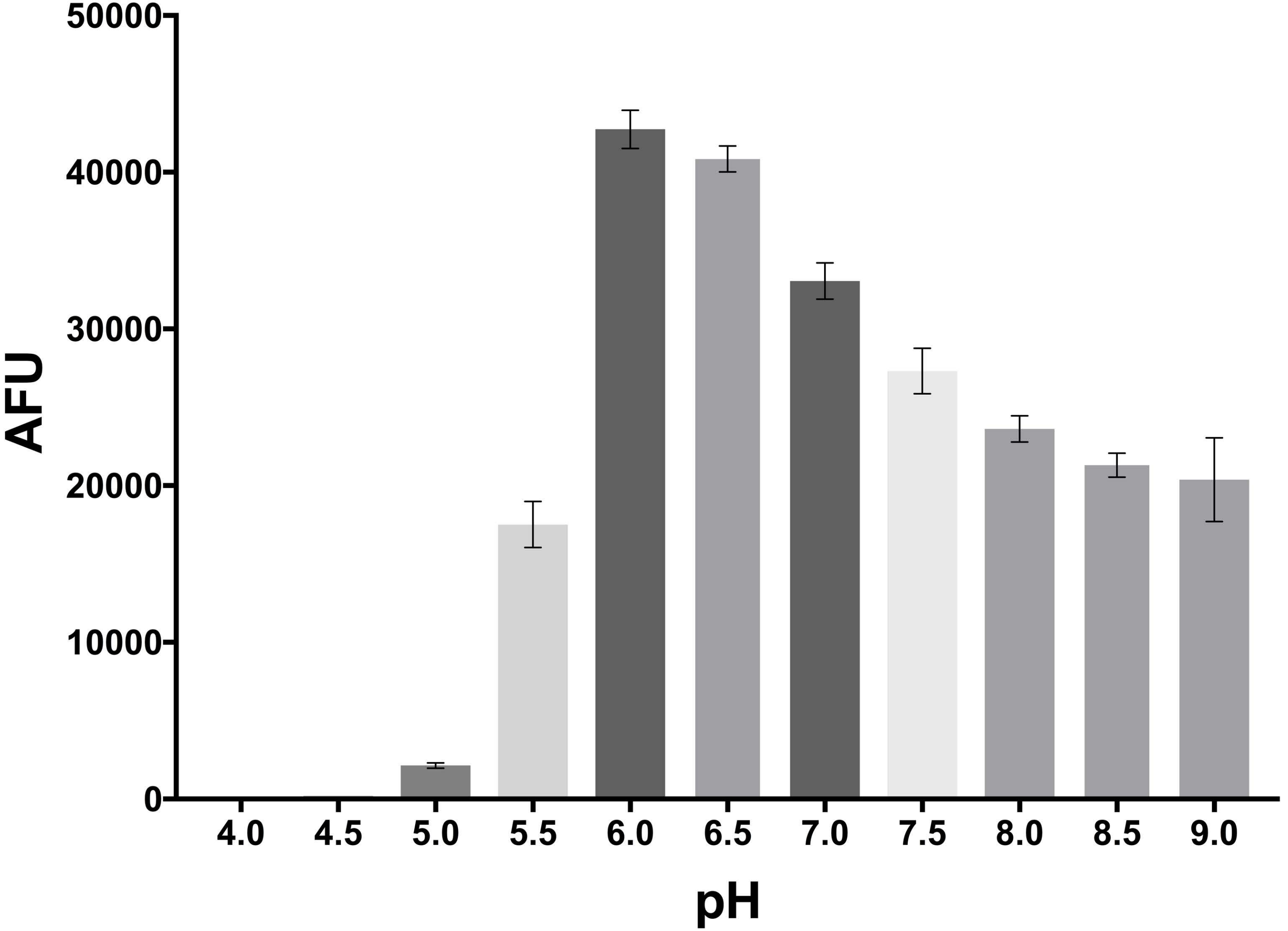
50000
40000
30000
20000
10000
0
AFU
4.0
4.5
5.0
5.5
6.0
6.5
7.0
7.5
8.0
8.5
9.0
pH

Supplemental Data:

Figure S1: Flow Cytometry Data Analysis. (a), BFP positive ECs were determined by selecting live cells by physical parameters (FSC and SSC). The gate for blue fluorescent positive cells was set such that a control population of untransfected cells was 0.5% BFP positive ECs. Individual comparisons of MG2P fluorescence in BFP negative (red) and BFP positive (blue) cells in each concentration of MG2P dye employed; (b) 0 nM, (c) 10 nM, (d) 50 nM, (e) 100 nM and (f) 500 nM.

Figure S2: MMP14 biosensor and MG2P dye co-localize in 3D. (a-c), Whole-cell image overlays showing colocalization imaging of HUVECs expressing the MMP14 biosensor, MG2P dye, and a fluorescent marker for either the endoplasmic reticulum (ER; a), the Golgi-apparatus (b), or lysosomes (c). (a'-d'), xz and yz panels of z-stack images of ER (a, c'), Golgi (b, c''), and lysosomes (c, c'''). Scale bars = 10 µm (whole cell); 5 µm (xz and yz panels).

Figure S3: The MMP14 biosensor is not pre-cleaved. (a, b), Whole-cell image overlays (far upper-left images) and zoomed regions of the cell (yellow boxes) showing colocalization imaging of HUVECs expressing the MMP14 biosensor, cell-permeable MG ester dye, and a fluorescent marker for either lysosomes (LAMP1) and exocytic vesicles (Rab8a). (a), Dye-binding is present in lysosomal vesicles (yellow arrows) but not in Rab8a-labeled exocytic vesicles (yellow circles), highlighting that the biosensor is not cleaved prior to reaching the cell surface. (b), Dye-binding is not detected in Rab8a-labeled, MMP14 enzyme containing exocytic vesicles.

The color of the text in each panel indicates the color of each label shown in the overlay images. (c), Quantification of the total number of MMP14 biosensor and MG ester (n = 7) or MG2P (n = 14) dye-bound biosensor and their distributions in lysosomes (n = 7), in Rab8a exocytic vesicles (n = 7), or in MMP14 overexpressing vesicles (n = 6). Scale bars = 20 µm (whole cell); 5 µm (zoomed). Error bars = +/- SD.

Figure S4: MG2P dye binding to MMP14 biosensor in different cell types. (a-e), Confocal images showing MMP14 biosensor (left panels), MG2P dye (middle panels), and Overlay (right panels). (a-b), HUVEC expressing MMP14 siRNA (b) displays reduced MG2P dye binding compared to a HUVEC expressing MMP14 biosensor alone (a). (c), MCF7 cell expressing the MMP14 biosensor alone displays no MG2P dye binding. (d), MCF7 cell expressing GFP-MMP14 (inset in d) displays increased dye binding to biosensor compared to (c). (e), MG2P dye binds MMP14 biosensor in MDA-MB-231 cells.

Figure S5: Fluorogen intensity is similar across a range of physiological pH. Endpoint fluorescence was measured after binding V_H fluorogen activating protein to MG2P. The experiment was run with RFUs normalized against the condition with the highest value (n = 3). The normalized average was plotted with error bars = +/- SD. AFU = Arbitrary Fluorescence Units.

Video 1: MMP14-Biosensor and MG2P dye trafficking near the plasma membrane (related to Figure 2).

Time-lapse video showing grayscale biosensor (left panel) and grayscale MG2P (middle panel), movements near the plasma membrane. Overlay (right panel) shows MMP14 biosensor (blue) and MG2P dye (pink). Total imaging time = 240 sec. Scale bar = 5 µm.

Video 2: MMP14-Biosensor and MG2P FRAP (related to Figure 3).

Time-lapse video showing FRAP of the grayscale biosensor (left panel) and grayscale MG2P (middle panel). Overlay (right panel) shows MMP14 biosensor (blue) and MG2P dye (pink). Total time = 367 sec. Scale bar = 5 µm.

Video 3: MMP14 activity in ECs is increased in the leading edge compared to the trailing edge (related to Figure 5).

Time-lapse video showing grayscale biosensor (left panel), grayscale MG2P (middle panel), and Overlay (right panel) in a polarized HUVEC migrating toward the top of the image window. Increased MG2P fluorescence intensity is seen within the leading edge (top) compared to the trailing edge (bottom). Total imaging time = 70 sec. Scale bar = 5 µm.

Video 4: HUVECs in 3D collagen I gel with biosensor and MG2P dye (related to Figure 5).

Z-stack images of a HUVEC cultured in a 3 mg/mL 3D collagen sandwich gel moving from the bottom of the cell to the top of the cell. The video shows a single time point Overlay composed

of collagen gel (DIC), MMP14-Biosensor (Blue) and MG2P-bound dye (Pink). Z step size = 0.2 μm; total Z = 5.8 μm. Scale bar = 5 μm.
